# Pain in Rheumatoid Arthritis: Could JAK Inhibition be the Answer?

**DOI:** 10.31138/mjr.31.1.112

**Published:** 2020-06-11

**Authors:** Puja Mehta, Peter C. Taylor

**Affiliations:** 1 Department of Rheumatology, University College London Hospital (UCLH), London, United Kingdom; 2 Nuffield Department of Orthopaedics, Rheumatology and Musculoskeletal Sciences, University of Oxford, Oxford, United Kingdom

**Keywords:** Rheumatoid arthritis, pain, JAK inhibition, JAK-STAT

## INTRODUCTION

Rheumatoid arthritis (RA) is an archetypal chronic, autoimmune inflammatory polyarthritis, which is characterized by peripheral joint pain, stiffness, and swelling. There have been considerable advances in management of RA, which has been revolutionized by the early and intensive use of immunotherapies. Despite these advances, pain is one of the largest unmet needs. Pain is multidimensional, defined by the International Association for the Study of Pain as “an unpleasant sensory and emotional experience associated with actual or potential tissue damage, or described in terms of such damage”.^1^ Patients with RA cite pain as their most important health priority.^2^ Pain has wide biopsychosocial implication and can increase work-disability and health-care utilization.^3^ Prognosis of pain in RA is often poor, even when inflammation is optimally controlled, ie, “remaining pain”.^4^ Better understanding of the characteristics, mechanisms and perception of pain, is critical in determining the most appropriate treatment approach. Escalation or switching of immunotherapies in patients with non-inflammatory pain, may be ineffective and unnecessarily risk loss of control of inflammation, as well as exposing patients to treatment-related adverse events. Furthermore, better understanding of non-inflammatory pain mechanisms may enable the development of targeted treatment strategies for specific subgroups of patients. The pain experience in RA is multifactorial, resulting from a complex interaction between genetics, psychology, comorbidities, joint pathology and alterations in both peripheral and central pain processing.^5^ Diagnosing, measuring and appropriately managing pain in RA is very challenging. There is accumulating evidence to suggest that targeting the Janus kinase/signal transducer and activator of the transcription (JAK-STAT) pathway may improve pain outcomes in RA. Here, we describe the impact, mechanisms and difficulties associated with measuring pain in RA and emerging role of JAK inhibition.

## THE BURDEN OF PAIN IN RA

Despite therapeutic advances and improved clinical outcomes, pain remains a considerable unmet need, which affects both quality of life and work capacity.^6^ Patients with RA commonly highlight pain as their most important problem, as demonstrated by a study of 96 patients who ranked pain as the most important out of 17 patient-related outcomes (PROs).^7^ 68% of RA patients rate pain as their highest priority for improvement, and 90% of RA patients rate pain as one of their top three priorities.^8^ In an international observational study of PROs, the majority of the cohorts with established RA in Europe (60%) and the US (65%) reported discontent with pain management^9^. Contemporary unmet needs in RA are changing; health domains of pain, fatigue and mood disturbance are closely linked and associated with restrictions in social participation.^10,11^ In distinction to past generations of RA patients, where joint deformity and consequent disability were very evident to the treating physician, these contemporary unmet needs are of a subjective nature, and known only to the patient themselves.^11^ However, key treatment goals of physicians are achieving remission, reduction in inflammation, prevention of structural damage and disability.^12,13^ It is therefore important that the treating physician recognises this dichotomy and having identified the issues that concern an individual patient, address them where possible with both pharmacological and non-pharmacological interventions as appropriate.

Pain is associated with high disease activity^14^ and can be reduced by early effective treatment of inflammatory disease.^15^ Female sex^14,16^ may be related to worse pain over time, and psychological factors influence pain reporting in RA^14,17^ and radiographic changes may be linked to future pain.^14^ Pain scores of patients with early severe rheumatoid arthritis are correlated with higher levels with patients’ global assessment of disease, morning stiffness and to a lesser degree disability (measured by Health Assessment Questionnaire tool; HAQ and C reactive protein; CRP) rather than with radiographic changes.^18^

## MECHANISMS OF PAIN IN RA

Clinically, RA is identified by synovitis, which classically corresponds with inflammation-driven pain. Studies have shown that inflammation of the synovium is accompanied by prostaglandin and bradykinin production, which leads to the activation of thin unmyelinated sensory nerves (C fibers) in the synovium^19^. The development of generalized and widespread pain in RA may be in large part related to the inflammatory impact on the peripheral nerves.^20^ Thus, inflammatory actions on nerve endings, including nociceptive fibres, may result in long-term sensitization, which contributes to chronic pain conditions. Proinflammatory cytokines like tumour necrosis factor (TNF) and interleukin 6 (IL-6) are both of specific importance in RA pathogenesis, and cytokine blockade is therapeutically beneficial. Both TNF and IL-6 also affect pain thresholds in experimental arthritis,^21,22^ as well as long-term sensitisation of joint nociceptors.^19^ It is postulated that arthritic joints expand their total receptive field to the surrounding area of normal noninflamed tissue. The enhanced response to stimulation of joints that are inflamed could be mediated by peripheral sensitization.^23^ Additional increased pain responses to noninflamed tissue could be generated in the spinal cord, leading to central sensitisation. Central sensitisation occurs when normal inputs begin to produce aberrant feedback due to the excitation of the neurons in the central nervous system. Studies have shown that anxiety, low mood, and depression have an impact on clinical pain reporting in RA and can influence brain activation in frontal regions, measured by brain functional neuroimaging studies.^24^ Interestingly, patients with RA treated with biologic therapies were found to have early improvements in brain pain sensitization, observed by brain functional imaging using blood oxygen level-dependent signals, within days of treatment with biologic agents, predating an observed clinical response in peripheral joint synovitis.^25^

It is thought that central sensitization to nociceptive stimuli results in a decrease in the threshold (allodynia) and an increase in the responsiveness (hyperalgesia) to noxious stimuli. In RA, general hyperalgesia to mechanical and thermal stimuli have been reported,^26^, and decreased pain thresholds over nonpainful areas have been shown in both early^27^ and established^28^ RA. It is unclear when central sensitisation patterns are established in the disease course of RA, or if they are reversible or amenable to specific targeted therapy. It is important to remember that central sensitisation may result from sustained nociceptive input from joint inflammation or from comorbid conditions such as fibromyalgia, secondary osteoarthritis, chronic lower back pain, obesity, smoking and diabetes.

## PAIN AND DISEASE ACTIVITY SCORES (DAS)

Pain is considered to be one of the cardinal features of inflammation and therefore physicians have historically considered pain a marker of disease activity. Current management recommendations emphasise a treat-to-target approach to achieve remission, in which treatment is titrated according to composite measures of disease activity. The DAS28 is comprised of tender (TJC28) and swollen joint count (SJC28) out of a defined set of 28 joints, a patient self-reported global health evaluation assessed on a visual analogue scale (VAS-VAS-GH), and an acute phase marker (CRP or ESR [Erythrocyte Sedimentation Rate]). A DAS28-ESR >5.1 is the threshold used to determine high-disease activity and biologic eligibility in the UK; a DAS28-ESR ≥3.2–5.1 indicates moderately active disease, and <2.6 disease remission. Of these components, SJC28 and acute-phase marker are observed by the assessor, and VAS-GH and TJC28 are reported by the patient.

Non-inflammatory pain (and its effects on the TJC and VAS-GH) can confound interpretation of the DAS28-ESR ≥3.2 as a measure of active inflammation. Non-inflammatory pain can result in misclassification of patients in remission as having active inflammation leading to inappropriate escalation of treatment and can compromise clinical trial inclusion criteria and outcomes. These scores also serve as regulatory efficacy endpoints for drug-licensing and are commonly criticised for not fully capturing the patient-experience or being sensitive enough to personalise treatments.

### Confounders of DAS28

Self-reported disease activity and joint tenderness on palpation may increase with inflammation from active RA, but may also be increased by changes in pain processing (centrally augmented pain) or comorbidities. TJCs and the VAS-GH were high in patients with established RA fulfilling fibromyalgia (FM) classification.^29^ Patients with fibromyalgia (without RA) generate high DAS28 scores comparable to patients with active RA, mainly due to high reporting of TJC, despite low SJC and acute phase markers.^30^ Central sensitisation and joint inflammation may coincide in patients with confirmed RA; patients with RA and comorbid fibromyalgia report increased pain^31^ and have higher DAS28 scores^32^ than those with RA alone, suggesting that central sensitisation influences the score. It is unclear if fibromyalgia is a true co-morbidity of RA, or whether this represents a mechanism of pain processing as part of RA. Other symptoms such as anxiety, depression and fatigue are strongly associated with chronic pain, may influence the DAS28, and might also reflect overlapping mechanisms within the central nervous system in people with RA.^5^

### Measuring non-inflammatory pain using DAS28

Pain is a difficult symptom to measure because it fluctuates and may be experienced or described differently by people at different times (inter- and intra- patient variability).

There have been several attempts to measure non-inflammatory pain contributions using the components of the DAS28-ESR. The absolute difference in tender and swollen joint has been shown to be associated with FM status (“fibromyalgic RA”) in a population of RA patients.^33^ Data from a Swedish national registry suggested that the ratio of tender to swollen joint might predict a reduced treatment response of RA to biologic therapy.^34^ The DAS28-P is the proportion of DAS28-ESR attributable to patient-reported components (TJC and VAS-GH) and is only calculated for active RA cases due to loss of normality at low values of DAS28.^35^ The DAS28-P index is a derived index, calculated using the following equation: (0.56 × √TJC)+(0.014 × VAS-GH) / (0.28 × √SJC) × (0.56 × √TJC) + (0.7 × ln(ESR)) + (0.014 × VAS-GH).^35^. Higher baseline DAS28-P in early RA (in the Early Rheumatoid Arthritis Network (ERAN) inception cohort) predicted less improvement in pain at 12 months.^35^ DAS28-P was associated with pain severity and predicted future pain in early^35^ and established^36^ RA and in patients commencing or changing biologic or non-biologic disease-modifying antirheumatic drugs (DMARDs).^36^ The DAS28-P has been associated with increased pain sensitivity and concurrent FM in people with RA.^29,37^ However, the DAS28-P may have limited utility when the DAS28 is <3.2, as small denominators used in its calculation would likely lead to high measurement error.

A recent study demonstrated the utility of derived indices as measures of non-inflammatory mechanisms of pain in patients with active RA. The authors obtained data from multiple observational epidemiology studies of people with active RA (DAS28>3.2), using baseline and follow-up data from the BSRBR participants (1) commencing anti-TNF therapy (n=10,813), or (2) changing between non-biologic DMARDs (n=2992), (3) Early Rheumatoid Arthritis Network participants (n=813), and (4) participants in a cross-sectional study exploring fibromyalgia and pain thresholds (n=45).^27^ Derived indices were the proportion of DAS28 attributable to patient-reported components (DAS28-P), tender-swollen difference and tender:swollen ratio. CRP was used as a co-variate marker for inflammatory disease activity. Pressure pain detection threshold (PPT) at a distance from affected joints was used as an index of central sensitisation. DAS28, TJC, VAS, DAS28-P, tender-swollen difference and tender:swollen ratio were more strongly associated with pain, PPT and fibromyalgia status than were SJC or ESR. DAS28-P, tender-swollen difference and tender:swollen ratio better predicted pain over 1 year than did DAS28 or its individual components. With respect to other PROs, DAS28-ESR and its components and each of the derived indices (including DAS28-P) were significantly associated with SF-36 Bodily Pain scores in each participant group. There were no significant associations between SJC or ESR and PPTs or FM classification, suggesting that ongoing inflammation might not be necessary to sustain central sensitisation in RA, but may have contributed to the development of sensitisation earlier in the disease course. Self-reported fatigue and poor mental health scores are associated with worse pain and greater central sensitisation in people with RA. Fatigue and/or poor mental health might, therefore, be additional centrally mediated non-inflammatory mechanisms that confound inflammatory disease assessment using DAS28-ESR. Limitations of this study include using the CRP as a sole measure of inflammation, as some patients with RA have ongoing inflammation, despite a normal CRP and the absence of imaging (MRI or ultra-sound) to evaluate subclinical synovitis.

### Remaining/Residual Pain

In early arthritis, it is thought that active joint inflammation causes a significant burden of pain. However, several studies show that pain may persist in inflammatory remission.^4,38^ Multiple randomized controlled trials have reported significant pain reduction associated with treatment with DMARDs, but many patients still experience clinically meaningful levels of remaining pain despite treatment.^39^ A minority of patients attain and sustain remission.^9,40^ However, even in patients achieving remission, residual pain may be present in 28%, suggesting that not all experiential pain is inflammatory in origin.^9,39^ A study from the British Society for Rheumatology Biologics Register (BSRBR) reported that bodily pain scores improved in both RA patients started on biologic DMARDs and non-biologic DMARDs.^36^ However, after 1 year of treatment, pain scores in both groups continued to be greater than 1 standard deviation, worse than the general population average. These patterns were noted even among individuals with moderate to good responses to DMARD treatment, assessed by the European League of Rheumatism (EULAR) response criteria, EULAR remission criteria, and absolute values of SJC and ESR. In a study of DAS28 remission in the Arthritis and Rheumatology Clinic of Kansas and the RA Evaluation Study databases, mean and median pain scores were 2.43 and 1.50 on a 0-to-10 visual analogue pain scale.^41^ Although these values were relatively low, they were higher than the cut point of 1.25, which separated patients who were satisfied with their health from those who were not. This observation indicated that, even among patients in DAS28 remission, many still have enough pain to negatively affect health satisfaction.

One study demonstrated that clinically significant pain continues among a substantial proportion of patients in DAS28 remission, but not among those in ACR/EULAR remission.^4^ Among 157 patients (out of a total of 865 assessed) in sustained DAS28-CRP remission for longer than 1 year, the prevalence of clinically significant pain (MDHAQ pain ≥4) was 11.9% at baseline and 12.5% at 1 year. No markers of inflammatory activity were associated with increased pain severity at baseline or 1 year. Patient global assessment, disability (MDHAQ function), fatigue (MDHAQ fatigue), sleep problems (MDHAQ sleep), and self-efficacy were strongly associated with pain at both baseline and 1 year. Inflammatory disease activity and joint damage (assessed using sharp scores) were not significantly associated with MDHAQ pain at baseline or at 1 year. Fatigue, sleep problems, and poor self-efficacy are part of a noninflammatory symptom cluster associated with “symptom intensification” syndromes, which may result from deficits in central sensitisation, resulting in allodynia and hyperalgesia. Disease duration was also significantly associated with pain severity at 1 year, which may reflect accrual of joint damage with time or progression towards a hyperalgesic state. Disease duration, however, was not associated with pain severity at baseline, suggesting that the observation may have been due to chance or a fibromyalgic state that was present at one year, but not at baseline. Clinically significant pain was not seen in patients with ACR/EULAR remission, likely due to the ACR/EULAR criterion limiting the patient global assessment score to ≤1. As the patient global assessment is heavily influenced by pain, this criterion essentially excludes patients with high pain scores. This study was limited by the lack of assessments involving the feet, subclinical synovitis and racial heterogeneity (which may influence pain perception). The authors discussed the difficulty in evaluating change in pain levels. They used a median improvement in pain of 20% in logistic regression models as a threshold for determining improvement; however, a clinically meaningful improvement threshold is unclear.

A study of 1241 unselected, early RA patients from the Swedish registry demonstrated that 58% patients had remaining pain (pain >20mm using a 100mm VAS) in spite of a good EULAR response at 3 months of treatment with methotrexate (MTX) monotherapy.^39^ Remaining pain, in spite of good EULAR response, was associated with higher baseline disability, using the HAQ (adjusted OR 2.2; 95% CI 1.4–3.4, per unit increase) and less baseline inflammation, using ESR (adjusted OR 0.81 [95% CI 0.70–0.93] per 10-mm increase). In addition, higher baseline VAS-GH associated with remaining pain, but markers of ‘inflammation’ (SJC28, CRP level) and TJC28, current smoking, RF and anti-CCP antibody positivity were not associated with remaining pain. Similar associations were detected for remaining pain at follow-up, despite low inflammatory activity defined as a CRP <10 (37% of all patients). In this group, remaining pain was significantly associated with baseline HAQ (OR 1.45 [95% CI1.17–1.79]), VAS-GH (adjusted OR 1.1 [95% CI 1.05–1.16]), and TJC28 (adjusted OR 1.04 [95% CI 1.01–1.06]), but not with baseline ESR (OR 0.86 [95% CI 0.81–0.91]), adjusted for age and sex, or CRP level (adjusted OR 0.83 [95% CI 0.79–0.88]). This suggests that patients with low inflammation at baseline may have a reduced ability to respond to DMARD treatment with respect to pain. Furthermore, baseline pain and VAS-GH and lower baseline ESR and CRP were predictive for remaining pain, suggesting that a substantial proportion of patients may already have a widespread pain syndrome at diagnosis, which is unlikely to respond to anti-inflammatory, immunosuppressive therapy. Patients with remaining pain, also displayed significant reductions of ESR, SJC28 and TJC28 at three months, indicating the presence of a reversible joint inflammation at diagnosis. CCP and RF status in patients with remaining pain and good EULAR response was similar, suggesting that a misclassification of fibromyalgia and RA was unlikely. Although subclinical synovitis was not directly assessed in this study, baseline pain was low in the subgroup with remaining pain; the converse would have been expected if there was subclinical inflammation. This study suggested that patients who develop remaining pain, may have been sensitised by initial low-grade inflammation on nociceptive fibres. Almost 20% patients had an increase in pain over the three month follow-up period, predicted by low baseline VAS-VAS-GH and low TJC28, suggesting the development of fibromyalgia or central sensitisation throughout the disease course (rather than at diagnosis) or poor pain coping strategies. The study was limited by the lack of assessment for subclinical synovitis, depression/anxiety and short treatment duration, as 12 weeks may be too short a duration to observe the full treatment effect of an immunosuppressive therapy.

Most disease-modifying therapies may exert benefit by suppressing inflammation, but may not improve or reverse dysregulated pain thresholds, hyperalgesia or allodynia. Of note, similar patterns have been shown in models of transient experimental arthritis, where initial joint inflammation was followed by a long-lasting pain behaviour, persisting after resolution of inflammation and non-responsive to anti-inflammatory medication.^42^

### painDETECT in RA

The painDETECT questionnaire was originally designed using chronic lower back pain patients and is used as a surrogate marker for the presence of neuropathic pain and central sensitisation. It has not been validated in RA, but studies have demonstrated its utility. In a study of 100 RA patients with well-controlled disease (mean DAS28 2.07+/−0.9), 28% had possible neuropathic pain and 5% had features of likely neuropathic pain by painDETECT scoring.^43^ There was a positive correlation between VAS and painDETETCT (r^2^=0.757). Obese patients were more likely to report pain than subjects with a normal BMI. Mood disorders (likely to influence pain) were not evaluated in this study. In the DANBIO Danish registry of 3,826 RA patients, 20% of patients had neuropathic pain using painDETECT scoring, which was associated with DAS28-CRP and VAS, but not with indicators of peripheral inflammation (CRP and SJC).^44^ In the prospective FRAME-cohort 102 RA patients were studied using clinical assessments, MRI hand imaging and PROs, including painDETECT, in order to evaluate the prognostic value of pain classification by the painDETECT score in relation to change in DAS28-CRP, VAS pain, and RAMRIS (MRI) score in RA patients initiating or escalating anti-inflammatory treatment.^45^ No prognostic value of painDETECT pain classification was found in relation to change of DAS28-CRP, RAMRIS score, or VAS pain. Intriguingly a high painDETECT score (non-nociceptive pain) at baseline was not associated with worse outcomes, in fact these patients had numerically greater improvement in DAS28-CRP, ie, reversible inflammatory driven pain hypersensitivity. Pain classification by painDETECT was not independently associated with change in DAS28-CRP, RAMRIS score, or VAS pain in the prognostic models. Patients with unclear pain mechanisms had reduced numerically treatment response, suggesting that their pain experience had been uncoupled from inflammation.

### Measuring pain

Articular or peri-articular inflammation may cause pain. The presence of non-inflammatory pain mechanisms does not exclude concurrent inflammation, and even moderate disease activity is longitudinally associated with both poor function and joint damage,^46^ which may in turn lead to mechanical reasons for pain. Assessment for subclinical synovitis with ultrasound or MRI is important in spite of DAS28 remission, and may help to titrate treatment to achieve remission, especially where non-inflammatory mechanisms might conceal contributions of persistent synovitis to DAS28-ESR.

Non-inflammatory mechanisms underlying important symptoms such as pain, fatigue, depression or anxiety are important when patients have persistent pain, despite the DAS28-ESR suggesting that inflammation is well-controlled. Derived measures (such as the DAS28-P) are unlikely to be appropriate for patients in DAS remission, where more experimental measures such as PPT or other forms of quantitative sensory testing might reveal non-inflammatory pain mechanisms.

There are no clear accepted definitions of thresholds for pain and remaining pain. Traditionally studies have evaluated pain improvement using ‘change from baseline’ measures. A recent post-hoc analysis of the RA-BEAM trial focussed specifically on pain outcomes.^47^ In RA-BEAM, a Phase 3 clinical trial of baricitinib, an oral, selective inhibitor of JAK1 and JAK2, baricitinib plus methotrexate (MTX) was associated with significant clinical improvements compared to patients treated with adalimumab plus MTX or placebo plus MTX. Baricitinib- and adalimumab-treated patients demonstrated similar improvement in SJC, with both groups demonstrating statistically significantly greater improvement relative to the placebo group beginning at Week 1 that was maintained through the placebo-controlled period (Week 24). For patient-reported pain, however, baricitinib-treated patients reported statistically significantly greater improvements as early as Week 1 compared to placebo-treated patients, and as early as Week 2 when compared with adalimumab-treated patients.

In the absence of standard pain thresholds in RA, the authors took two approaches. First, they applied percent change from baseline threshold recommendations from the chronic pain literature from the Initiative on Methods, Measurement, and Pain Assessment in Clinical Trials (IMMPACT), an organisation which aims to improve clinical trials of treatments for pain.^48^ A 30% improvement threshold is described as “much improved, meaningful differences” and 50% represents “very much improved, substantial improvement” in chronic pain conditions. A 70% improvement threshold, although not defined in IMMPACT, was also evaluated, because it is analogous to ACR response endpoints. To evaluate absolute pain, the authors studied thresholds of remaining pain (ie, the absolute value of patient-reported pain) of ≤10 mm, ≤20 mm, or ≤40 mm, at Week 24. The ≤20 mm threshold indicates when satisfaction with health is not negatively affected by pain, and was the cut-off selected for ‘remaining pain’. The ≤40 mm threshold was derived from observed cut-off points between the pain VAS and 122 the Patient Acceptable Symptom State (PASS). A mediation analysis with multiple mediators was conducted to evaluate the relationship between levels of inflammation and pain relief. The total treatment effect on pain relief over placebo, that could be accounted for by changes in CRP, ESR and SJC in the mediation analysis, was the ‘indirect’ or mediation effect, whilst the total treatment effect remaining was termed the ‘direct’ effect.

Baricitinib demonstrated greater and more rapid achievement of clinically significant levels of pain relief than adalimumab or placebo through Week 24. Furthermore, this differential effect became more marked as the pain relief thresholds increased, with approximately 40% of the patients receiving baricitinib achieving ≥70% pain relief from baseline by Week 24.^47^

Intriguingly there was a very rapid onset of effective mean pain relief at a cohort level with baricitinib and MTX.^47^ For those patients achieving ≥50% or ≥70% pain relief, baricitinib had a shorter median time to achieving these pain relief thresholds than placebo or adalimumab. Specifically, for ≥50% pain relief, the 4 weeks needed for baricitinib was approximately half that of adalimumab treated patients (8 weeks). For patients achieving ≥30% pain relief, baricitinib and adalimumab had similar median time to onset (approximately 2 weeks). Interestingly, the differences in pain relief between baricitinib and adalimumab could not be accounted for by effects of inflammation, as measured by CRP.^47^

These findings are also demonstrated by tofacitinib, which blocks JAK 1 and 3, and is the most extensively studied JAK inhibitor. There is significant reduction in RA patients’ assessment of pain with tofacitinib compared to placebo. Some patients reported pain relief within the first 24 hours of JAK inhibitor administration, well before a demonstrable effect on inflammation.^49^ Tofacitinib, administered 5 mg bd, was associated with a 45%–54% improvement in the patients’ assessment of pain and a 44%–60% improvement in Physician Global Assessment (PGA) score, while placebo resulted in less improvement (29% for pain and 39% for Physician Global Assessment [PtGA]).

## THE JAK-STAT PATHWAY IN PAIN MODULATION

The JAK-STAT intracellular pathway, is involved in a complex cytokine cascade, generating both pro-nociceptive as well as pro- and anti-inflammatory cytokines.^50^ Pro-inflammatory cytokines that may contribute to hyperalgesia include TNF-α, IL-6, IL-4, IL-1β, IL-12, IL-18 and granulocyte macrophage colony stimulating factor (GM-CSF).^47,50^ Of these, all but TNF-α, IL-1β and IL-18 signal via the JAK-STAT pathway. IL-6 induces JAK (JAK1 and JAK2) mediated phosphorylation of STAT1 and STAT3 proteins. IL-6 and STAT3 are key mediators of both chronic inflammation and joint destruction in RA. In the CNS, STAT3 plays an essential role in IL-6 signalling. The blockade of the JAK-STAT3 activity prevented the strong expression of IL-6 and of other factors induced in the spinal cord after nerve lesion in rats and attenuated mechanical allodynia.^51^ GM-CSF signals through JAK2 homodimers. In a rodent collagenase-induced instability model of osteoarthritis, pain was shown to be GM-CSF dependent and neutralising GM-CSF rapidly and completely abolished arthritis pain.^52^

## CONCLUSIONS

Pain represents a significant unmet need in managing many rheumatic conditions and results from a complex interaction of structural damage, inflammation, peripheral sensitisation and central amplification. The observation of JAK inhibitors ameliorating not only inflammation but also pain in patients with RA is very promising. Although the exact mechanisms of pain modulation associated with the JAK-STAT pathway is unknown, multiple, simultaneous cytokine blockade with JAK inhibitors, is likely to play a key role. Further investigation into the mechanistic basis for pain relief and the impact on suffering associated with JAK inhibition is warranted. In time, clinicians will have better tools to personalise therapies according to the individual pain profiles/signatures to improve the outcome for patients.
